# Patient safety of adjunct pre-operative intravenous S-ketamine for pain relief in third molar surgery – a randomised, placebo-controlled, double-blind trial

**DOI:** 10.1177/20494637241262509

**Published:** 2024-06-20

**Authors:** Lars B Eriksson, Torsten Gordh, Rolf Karlsten, Andreas Thor, Åke Tegelberg

**Affiliations:** 1 Department of Surgical Sciences, Uppsala University, Uppsala, Sweden; 2 Centre for Clinical Research, Uppsala University, Falun, Sweden; 3 Centre for Clinical Research, Uppsala University, Västerås, Sweden; 4 Department of Orofacial Pain and Jaw Function, Faculty of Odontology, 5264 Malmö University, Malmö, Sweden

**Keywords:** Adverse events, physiological effects, safety, S-ketamine

## Abstract

**Purpose:**

To study patient safety in third molar surgery, where two different doses of S-ketamine were administered for pain relief and compared to a placebo (saline). The primary focus was capillary oxygen saturation of the blood (SpO_2_) and secondarily, alterations in respiratory rate, blood pressure, pulse or adverse events.

**Methods:**

One hundred and sixty-eight subjects were included in a randomised, placebo-controlled, double-blind trial. The two subanaesthetic study drugs were low-dose S-ketamine (0.125 mg/kg) and high-dose S-ketamine (0.25 mg/kg). Every patient was sedated with midazolam prior to infusion of the investigational drug. The teeth were surgically removed according to a routine clinical procedure, under local anaesthesia.

**Results:**

Primary end-point for the safety aspects was capillary oxygen saturation (SpO_2_) after administration of the investigational drug was finished. A significant difference was found between the placebo and the high-dose group at that point (*p* = .021), with a decrease of saturation in the high-dose group. The lowest saturation and the number of registrations of SpO_2_ <90% did not show any difference between groups. Oxygen supplementation was given in circa 40% of the cases with no differences between the intervention groups. No other significant differences between groups regarding saturation or respiratory rate were noted.

**Conclusion:**

In this study, it was safe to use adjunct preoperative single-dose intravenous S-ketamine 0.25 mg/kg body weight for pain relief, in midazolam-sedated patients receiving third molar surgery. There were no serious adverse events or symptoms of overdose nor any clinically relevant effects on circulatory or respiratory parameters.

## Introduction

Surgical removal of the mandibular third molar tooth is a common treatment for several reasons, such as recurrent or continuous pain due to local infection or inflammatory conditions.^1,2^ Third molar surgery is a common model in the study of pain management.^3^ Over the past two decades, more than 20,000 such procedures have been undertaken annually in Swedish oral and maxillofacial surgery clinics.^4^ Patients presenting for removal of mandibular third molar are generally young and healthy.

Postoperative pain from oral and maxillofacial surgery is most frequent and intensive on the day of surgery,^5^ peaking approximately 5 h post-operatively.^6^ When the effect of the local anaesthetic wears off, effective pain relief by medication is advised. Thus, third molar surgery involving both hard and soft tissues is a suitable model for the evaluation of postoperative pain management.^7,8^ Various agents are used for post-operative analgesia. To prevent and relieve postoperative pain after third molar surgery, either paracetamol or ibuprofen, or a combination of both, are the most commonly used medicines for pain relief and are considered effective and safe.^9–11^

In some cases, the paracetamol and ibuprofen regime are insufficient and thus an analgesic that is more potent is needed. S-ketamine could be used to reduce peroperative and postoperative pain and increasing the interval to the first dose of postoperative analgesic medication.^12–14^ In our previously published article we demonstrated a significant lower reported pain by VAS in the high-dose group (S-ketamine 0.25 mg/kg bw) compared to low-dose (S-ketamine 0.125 mg/kg bw) and placebo (saline), during the first 24 h.^15^ We administered the investigational drugs as a slow intravenous bolus infusion (5–10 min). Ketamine is free from some of the, mainly respiratory, side effects related to opioid-analgesics.^16,17^ S-ketamine’s pharmacodynamics profile has been well known for decades. The drug’s characteristic features place it in a category of its own, and there is no similar drug currently in professional use. Developed for anaesthetic purposes, over the years ketamine has been found to be useful in several other applications.^18,19^ Ketamine causes analgesic, amnestic and psychosensory effects.^19^ Apart from its anaesthetic effect, ketamine is also a potent intravenous analgesic and is also used in various chronic pain applications.^18–20^ Adverse effects of ketamine are mainly cognitive impairment and psychotomimetic. As a result of recreational use, acute adverse events may also include dizziness, hallucinations, abdominal pain and vomiting.^20^ The S (+) ketamine enantiomer is approximately twice as potent analgesic and anaesthetic as the racemic mixture.^19^ Ketamine is a non-competitive antagonist of the NMDA-receptors (N-methyl-D-aspartic acid receptor). When binding to the NMDA-receptor ketamine prevents calcium ions to flowing through the canal.^18^ In addition to blocking of NMDA receptors ketamine may interact with a wide range of receptors and systems modulating the pain transmission pathways.^18^ A signature feature for ketamine is that it, in contrast with all other anaesthetics, maintains some cortical activity across the surface of the brain.^21,22^ The mechanisms are poorly known in detail but it appears to be multiple factors in a complex combination where ketamine seems to partly exert its effect via the opioid and monoaminergic systems.^18^ The effect of ketamine is a dissociative state by selectively disrupting the association pathways in the brain, inducing a catalepsy-like state that results in unconsciousness, amnesia and effective pain relief.^18,22,23^ The dissociative condition are dreamlike and vivid dreams of hallucinatory nature are often described.^24^

Ketamine is also active in reducing pain-related negative emotions that in rodent chronic pain models has been shown as reduced aversion, even after the expected analgesia duration. In combination with midazolam, psychotomimetic and dissociative symptoms such as dreamlike states, nightmares and hallucinations, that are known side effects associated with ketamine, can be reduced or eliminated.^25–28^

The anaesthetic and analgesic effects of S-ketamine are well known.^29–31^ To implement S-ketamine in routine day case, third-molar surgery we need to know its influence on basic physiological parameters. When assessing the risk/benefit, we also need to explore the significance of potential side effects in relation to the route of administration, doses and indication in this specific population.

Surgical removal of teeth can be unpleasant, frightening and can be the cause of anxiety. As a part of the treatment, benzodiazepine, such as a midazolam sedation, could reduce fear. When indicated the use of midazolam is a routine clinical method for that purpose.^32,33^ At therapeutic doses, peri-operative midazolam sedation has no significant effect on respiration or circulation,^34–38^ but may reduce ventilation in higher doses; however, there is a wide margin between effective doses and those associated with adverse events. In this current article, we present safety data from our previously published trial by analysing the results from secondary outcome variables.^15^

### Aim

The aim was to study patient safety in third molar surgery, with special reference to capillary oxygen saturation of the blood (SpO_2_) and secondarily alterations in respiratory rate, blood pressure, pulse or adverse events, all following administration of two different doses of S-ketamine, 0.125 mg/kg or 0.25 mg/kg, compared to placebo (saline) in midazolam sedation.

## Study design

A randomised, placebo-controlled, double-blind, consecutive study was performed. The regional ethics committee of Uppsala University, Uppsala, Sweden (Dnr 2015/378) and the Swedish National Medical Products Agency (MPA) (Dnr 5.1-2016-48439) approved the clinical study protocol, patient information sheet and informed-consent form. Before any study-specific procedures were undertaken, all patients signed the informed-consent form at the screening appointment. The study was monitored at four occasions by an independent observer and registered in both EudraCT (2014-004235-39) and ClinicalTrials.gov (ID: NCT04459377).

## Patients and methods

The subjects were referred to the Department of Oral and Maxillofacial Surgery at the County Hospital, Falun, Sweden for surgical removal of a mandibular third molar.

One hundred and sixty-eight subjects met the inclusion and exclusion criteria and accepted the invitation to participate in the study.

The inclusion criteria were healthy men and women or those with a well-compensated, mild systemic disease, according to the ASA I and II-criteria.^39,40^ Subjects were aged 18–44 years and had a body weight between 50 and 100 kg.

The exclusion criteria for subjects were reported hypersensitivity or allergy to midazolam, ketamine or ibuprofen and subjects who were prescribed daily medication such as analgesics, hypnotics, psychopharmaceuticals and/or MAO inhibitors. Other reasons for exclusion were pregnancy, breastfeeding, or conditions such as confirmed hepatitis B or C or HIV, myasthenia gravis and/or verified sleep apnoea and patients who were unable to comprehend written and/or spoken information in Swedish.

**Figure 1.**
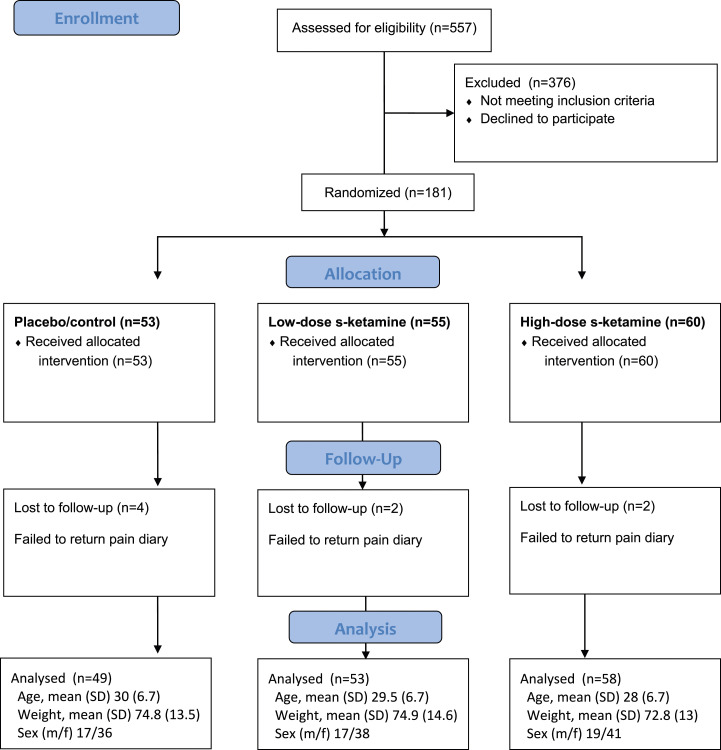
Consort 2010 flow diagram.

The use of inclusion and exclusion criteria was based on anamnestic data. No other forms of screening or assessment were made to verify the subjects’ history. A CONSORT flow-chart of the study design is shown in Figure 1.

### Methods

The two study drugs were low-dose S-ketamine (0.125 mg/kg) and high-dose S-ketamine (0.25 mg/kg), compared to placebo with saline solution. All subjects in these groups received conscious intravenous sedation.

The midazolam sedation served one main purpose, to reduce or eliminate nightmares and hallucinations related to S-ketamine.^20,28^ Every patient was sedated with midazolam prior to infusion of the investigational drug. In our previous article from this trial, we found the high-dose group (0.25 mg/kg bw) s-ketamine to score globally lower on visual analogue scale (VAS) for pain during the first 24 h postoperatively compared to low-dose group (0.125 mg/kg bw) s-ketamine and placebo group.^15^ The amount and timing of rescue pills did not differ significantly between the groups during the first 24 h postoperatively.^15^

### Procedure

The subjects were sedated to a defined endpoint: ptosis and/or dysarthria. At endpoint, the surgeon and an independent observer, according to the OAA/S-scale (Observer’s Assessment of Alertness/Sedation Scale),^41–43^ scored the depth of sedation. The OAA/S gradations (1: deep sleep to 5: awake) were applied in relation to each of the following factors: responsiveness, speech, facial expression and eyes. Two independent observers noted the lowest score of the four variables. The reason for the OAA/S scoring was to make sure that the depth of sedation was equal between subjects and over time. The whole calculated dose of 10–20 mL investigational drug were given to the subjects using a syringe pump at a pace of 2 mL/minute.

The teeth were removed according to a routine clinical procedure, using the local anaesthetic Xylocaine-adrenalin^®^ (20 mg/mL, 7.2 mL) [Dentsply DeTrey GmbH, Konstanz, Germany]. In cases of significant bleeding, haemostasis was ensured before closure of the wound with three simple loop absorbable sutures (Vicryl^®^) [Johnson & Johnson MedTech, New Brunswick, New Jersey, USA].

In order to minimise the number of unknown and undesirable factors, all operations were undertaken by the same surgeon (LBE).

Blood pressure, pulse rate as well as capillary saturation level (SpO_2_) was continuously monitored using infrared pulse oximetry (DASH 5000 monitor by GE Medical systems technologies, Nellcor algorithm) and noted in the case report form (CRF) preoperatively, at end of sedation, analgesia and surgery. Any desaturation event below SpO_2_ 90% was noted. The frequency of oxygen supplementation was also noted. Beginning right before surgery, patients completed a diary, where they recorded known side effects – such as nightmares, hallucinations, nausea or vomiting – by answering yes/no questions at the day of surgery and the day after surgery, and accounted for other adverse events experienced over the first 24 h, in free text.

The subjects were given ibuprofen 400 mg for potential use during the first post-operative day, at a recommended dose of one tablet 0–4 times a day, when needed for pain relief.^10^

### Safety and monitoring

The monitoring complied with guidelines except electrocardiography (ECG) and capnography coverage.^44,45^ During the surgical procedure, pulse, capillary oxygen saturation, SpO_2_ (continuously monitored) and non-invasive blood pressure (monitored every 5th minute) were automatically measured [DASH 5000 monitor by GE Medical systems technologies]. Respiratory rate was measured on four occasions: preoperatively, at the end of sedation injection, at the end of S-ketamine/placebo infusion and at the end of surgery. Benzodiazepine antidote (flumazenil) was available if needed, as well as oxygen for supplementation, in case of desaturation or in case there was a need for assisted ventilation; in cases of desaturation to approximately 90% SpO_2_ or lower, additional O_2_ was administered via a nasal cannula at 2–2.5 L/minute.

### Statistical methods

The basis of the power calculation was the pain by VAS 3 h after surgery – for details see reference.^15^ Based on the power analysis subjects were allotted to the groups (Table 1).

**Table 1.** Demographic and clinical characteristics of the included subjects.

	Group		
	Placebo	Low dose	High dose
	Saline solution	S-ketamine	S-ketamine
		0.125 mg/kg	0.25 mg/kg
No.	53	55	60
Sex			
Female	36 (68%)	38 (69%)	41 (68%)
Male	17 (32%)	17 (31%)	19 (32%)
Age, mean (SD), year	30 (6.7)	29.5 (6.7)	28 (6.7)
BMI, mean (SD)	25.4 (4.1)	25.5 (8.7)	24.7 (4.1)
Duration of surgery, minutes			
Median	15	16	14
IQR	9	9.5	12
Min	5	7	6
Max	70	35	48
Clinical degree of retention			
Erupted	2	3	1
Partially covered by mucosa	46	50	53
Entirely covered by mucosa	5	2	6
Entirely covered by bone	0	0	0
Radiological position			
Vertical	10	8	13
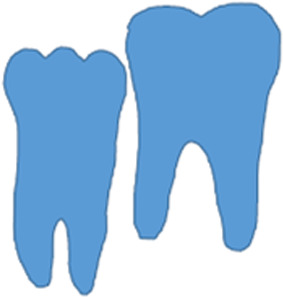			
Mesioangular	18	10	14
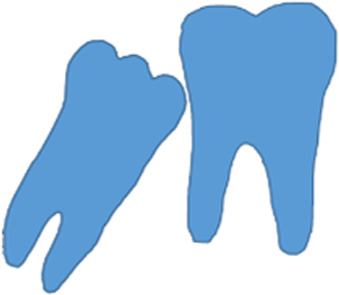			
Horizontal	14	18	13
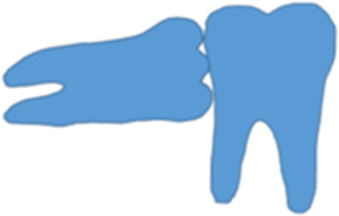			
Distoangular	11	19	20
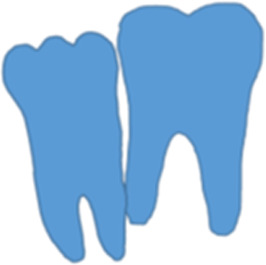			

Demographic and clinical characteristics.

Data were analysed due to the intention-to-treat principle, so therefore subjects were coded to their allocation status. For all our analyses, we used the software Jamovi, version 2.3.21. The comparison was conducted in a hierarchal procedure of predetermined order: (1) high-dose S-ketamine versus placebo and (2) low-dose S-ketamine versus placebo. The physiological variables SpO_2_, respiratory rate, pulse rate, systolic blood pressure and diastolic blood pressure were compared between groups using the independent non-parametric method, Mann–Whitney U test.

Fisher’s exact test was used to compare the groups regarding self-reported side-effects, such as any occurrence of nausea and vomiting on the day of surgery, nausea and vomiting the day after surgery, nightmares on the day of surgery and hallucinations on the day of surgery.

## Results

The mean operation time did not differ significantly between the groups (Table 1).

**Figure 2.**
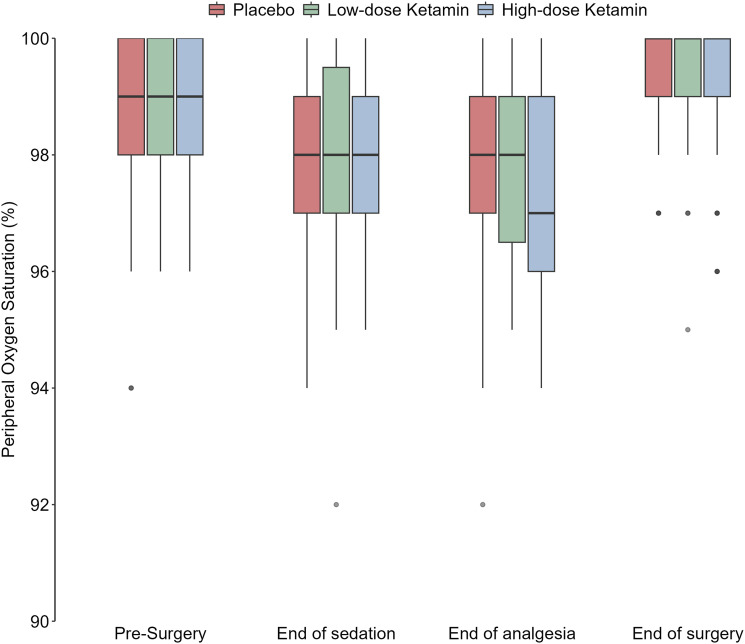
Capillary saturation (SpO_2_) at end-point.

For the safety aspects in this study, capillary oxygen saturation (SpO_2_) after finished administration of the investigational drug was the main outcome measure. There was a significant difference between the placebo and the high-dose group at that point (*p* = .021), with a decrease of saturation in the high-dose group (Table 2; Figure 2). No other significant differences between groups regarding saturation or respiratory rate were noted.

**Table 2.** Effects on respiratory parameters during the perioperative period.

Parameters and groupes	Baseline	End of sedation	End of analgesia (primary end-point)	End of surgery
Saturation* SpO_2_				
Placebo^1^	98.8 (1.4)	98.0 (1.7)	97.9 (1.7)	99.3 (1.0)
Low-dose^2^	98.9 (1.1)	98.0 (1.7)	98.0 (1.7)	99.3 (1.1)
High-dose^3^	98.8 (1.1)	97.8 (1.5)	97.1 (1.9)	99.2 (1.1)
p (1 vs 3)			0.021	
p (1 vs 2)			0.793	
Respiratory rate				
Placebo^1^	16.5 (3.1)	17.9 (2.7)	17.3 (2.5)	18.1 (2.9)
Low-dose^2^	16.3 (3.4)	17.0 (2.6)	16.8 (2.6)	17.4 (2.4)
High-dose^3^	16.4 (3.4)	17.6 (2.6)	16.7 (2.7)	17.5 (2.5)
p (1 vs 3)			0.308	

Placebo: saline; low-dose: 0.125 mg S-ketamine/kg; High-dose: 0.25 S-ketamine/kg. Mean (std). *Capillary saturation SpO_2_ [%].

Respiratory rate = breath/minute.

*p*-value based on Mann-Whitney U test.

The lowest capillary oxygen saturation and the number of registrations of SpO_2_ <90% did not show any difference between groups. Oxygen supplementation was given in around 40% of the cases with no differences between the intervention groups (Table 3).

**Table 3.** Frequency of oxygen supplementation. Oxygen supplemented thru a nasal cannula.

Group	*n*	Counts	%
Placebo	53	15	28.3
Low-dose (s-ketamine 0.125 mg/kg)	55	26	47.3
High-dose (s-ketamine 0.25 mg/kg)	60	25	41.7

At the end of the investigational drug administration, the pulse rate differed significantly between the placebo and the high-dose group (*p* = .007) with an increased rate in the high-dose group. No other differences between the groups regarding pulse rate or blood pressure were found (Table 4).

**Table 4.** Effects on haemodynamic parameters during the perioperative period.

Parameters and groups	Baseline	End of sedation	End of analgesia (primary end-point)	End of surgery
Pulse rate^a^				
Placebo^1^	70.1 (9.9)	71.7 (9.2)	70.3 (10.4)	86.3 (11.7)
Low-dose^2^	68.6 (12.3)	71.6 (10.4)	73.2 (11.8)	83.7 (14.0)
High-dose^3^	69.8 (13.3)	70.6 (10.2)	75.9 (10.8)	88.2 (12.6)
p (1 vs 3)			0.007	
p (1 vs 2)			0.155	
Systolic BP^b^				
Placebo^1^	116.6 (13.0)	106.0 (11.1)	103.8 (11.2)	117.2 (14.2)
Low-dose^2^	115.9 (13.1)	106.0 (11.3)	107.0 (14.3)	118.5 (14.7)
High-dose^3^	117.0 (13.1)	107.4 (10.4)	108.3 (13.7)	120.4 (15.9)
p (1 vs 3)			0.122	
Diastolic BP^b^				
Placebo^1^	69.4 (8.5)	63.2 (7.3)	62.0 (7.2)	63.5 (8.8)
Low-dose^2^	68.6 (9.2)	62.7 (7.9)	63.1 (10.1)	65.1 (8.8)
High-dose^3^	68.8 (8.1)	63.7 (7.6)	65.2 (9.5)	66.3 (9.9)
p (1 vs 3)			0.074	

Placebo: saline; low-dose: 0.125 mg S-ketamine/kg; high-dose: 0.25 S-ketamine/kg.

*p*-value based on Mann-Whitney U test.

^a^Frequency = bpm.

^b^mmHg. Mean (std).

Nausea and vomiting on the day of surgery were found to be more frequent in the high-dose group (*p* = .006), (Table 5). No other differences were found between all groups.

**Table 5.** Frequency of known side effects of ketamine.

	Groups			Fisher’s exact test *p*-value
	Placebo^1^	Low-dose S-ketamine^2^	High-dose S-ketamine^3^	
No	49 (29.1)	51 (30.3)	56 (33.3)	
Missing	4 (2.3)	4 (2.3)	4 (2.3)	
Nausea and vomiting day of surgery	1 (0.6)	7 (4.5)	12 (7.7)	.006 (1 vs 3) .060 (1 vs 2)
Nausea and vomiting day after surgery	2 (1.3)	2 (1.3)	6 (3.9)	.351
Nightmares day of surgery	1 (0.6)	1 (0.6)	4 (2.6)	.372
Hallucinations day of surgery	0 (0)	1 (0.6)	1 (0.6)	1.000

Placebo: saline solution; low-dose: 0.125 mg/kg; high-dose: 0.250 mg/kg. Number (%).

The subjects had the opportunity to describe any other complaints in a free text section. Sixty-two out of 168 subjects used that opportunity. Local problems at the site of surgery as well as pain that was not sufficiently relieved by the rescue pills were the most common complaints (Table 6).

**Table 6.** Summary of reported complaints in free text comments.

Complaint	Group			Total
	Placebo	Low-dose	High-dose	
Tiredness	3	2	3	8
Nausea/vomiting	4	4	0	8
Fever/algid	4	2	3	9
Headache	2	0	1	3
Hallucinations/dreams	2	0	1	3
Pain not cured by rescue pills	5	2	6	13
Panic/anxiety	1	0	0	1
Local problem associated with surgery site	7	5	6	18
Dizziness/fainting	2	5	0	7
Amnesia	1	1	1	3
Nightmares	0	0	0	0
Other	2	6	2	10
Total	33	27	23	

Frequency of complaints from free text section of patients’ pain diary.

83 complaints from 63 out of total 168 included subjects.

Placebo: saline solution; low-dose: 0.125 mg/kg S-ketamine; High-Dose: 0.250 mg/kg S-ketamine.

## Discussion

We found that intravenous S-ketamine 0.25 mg/kg bw (body weight) compared to placebo, leads to increased pulse rate and decreased capillary oxygen saturation and, also more frequent, nausea and vomiting during the day of surgery. All these changes are statistically significant but represented small differences of none or minimal clinical relevance. Even though our protocol was placebo controlled, all subjects received midazolam. Thereby all results mirror a combined effect of midazolam and ketamine. We have not found any descriptions of true interactions between midazolam and ketamine in the literature. In a study from 1993 about hypnotic and anaesthetic interactions in midazolam versus ketamine, the authors stated that there are additive hypnotic effects but found no evidence of interaction.^46^

Based on our findings, we consider it safe to use adjunctive, pre-emptive intravenous S-ketamine 0.25 mg/kg body weight in midazolam-sedated patients undergoing third-molar surgery. The slight decrease in saturation in the high-dose S-ketamine group at end of analgesia might be explained by a higher CNS (central nervous system) depressant effect than in the other groups. It may also be a sign of stronger analgesia that more effectively inhibits any nociceptive signalling from the periphery and therefore decreases the arousal effect on CNS, and indirectly, the respiratory drive. Ketamine might be seen as a dirty drug due to its omnipotent effect on receptors in the central and the peripheral nervous system. This fact could be a source of synergistic interaction with midazolam that may cause a respiratory depression and lowered SpO_2_, but no support has been found in the literature for that explanation.

There are several known side effects to ketamine use. In this study, set-up risks related to the single dose differs from repeated dosage as well as from recreational use/abuse and high doses. In repeated dosage and high dosage there are potential risks for serious and potentially persistent side-effects. Cognitive changes, urological and hepatic impact are among the recognised risks, as well as the risk of developing a dependence.^47,48^ Persistent and recurrent schizotypal behaviour has also been observed in recreational/abuse users.^49^

There is no significant reported risk for nausea or vomiting related to S-ketamine when used as an adjuvant in general anaesthesia.^50^ However, in the present study, postoperative nausea and vomiting (PONV) was more common on the day of surgery in subjects who received high-dose S-ketamine compared to low-dose and placebo.

In a study in children going through gastrointestinal endoscopies, the authors found that the procedural sedation with ketamine, with or without midazolam was safe and effective.^28^ In a large review about procedural sedation of children using ketamine, one main finding was the low incidence of adverse events (AE).^51^ In another study, occurrence of recovery agitation was shown to have been reduced in adults in emergency departments when they are given ketamine combined with midazolam compared to those who only got ketamine.^52^

All patients expect an effective treatment for their conditions. The less serious the condition is, the safer the treatment has to be. In other words, in conditions that are considered to be relatively harmless or pose a small threat to the person´s health, there is a very low acceptance of treatments or methods that could be harmful or pose a serious threat to the subject’s health. Any treatment or method must therefore be safe and not associated with risk of serious adverse events or unpleasant side effects. When using approved drugs such as ketamine that has been in clinical use for decades, most side-effects including rare side-effects are already known because of years of reporting adverse events. In a recent review article that included 3756 participants treated with sub-anaesthetic doses of ketamine for psychiatric disorders, the incidence of MSAE (medical serious adverse events) was approximately 0.1% of individuals.^53^

The cumulative mortality risk associated with anaesthesia for outpatient oral and maxillofacial surgery is 1:835 000.^54^ As a rule of thumb, the risk of death associated with anaesthesia for outpatient oral and maxillofacial surgery is approximately ‘one in a million’.^55^ We assumed that the risk associated with conscious sedation would be even less compared to general anaesthesia using higher doses over a longer time period.

## Conclusions

In this study, we found it was safe to use adjunct pre-emptive intravenous S-ketamine 0.25 mg/kg body weight for pain relief in midazolam-sedated patients receiving third-molar surgery. There were no serious adverse events or symptoms of overdose nor any clinically relevant effects on circulatory or respiratory parameters. These findings add to our previously published original findings of analgesic efficacy.^15^
